# Mapping the Expression Profile of MicroRNA in Peri‐Implantitis: A Scoping Review

**DOI:** 10.1155/ijod/7830337

**Published:** 2026-10-21

**Authors:** Madhumitha Muruganandam, Sangeeta U. Nayak, Ashita S. Uppoor, Arpit Gupta, Santhosh Kumar

**Affiliations:** ^1^ Department of Periodontology Manipal College of Dental Sciences Mangalore Manipal Academy of Higher Education Manipal India manipal.edu; ^2^ Department of Periodontology Manipal College of Dental Sciences Manipal Academy of Higher Education Manipal India manipal.edu

**Keywords:** dental implants, microRNA, miRNA, peri-implantitis

## Abstract

**Background:**

Peri‐implantitis remains a major biological complication compromising implant longevity. Conventional diagnostic tools, including clinical probing and radiographic evaluation, are limited in detecting early disease activity. MicroRNAs (miRNAs) have emerged as potential biomarkers due to their regulatory role in inflammation and bone metabolism.

**Objective:**

This scoping review aimed to map the available evidence on miRNA expression profiles in peri‐implantitis, identify knowledge gaps, and highlight miRNAs with potential diagnostic and therapeutic relevance.

**Methods:**

The review was conducted in accordance with Arksey and O’Malley’s framework, Joanna Briggs Institute guidelines, and PRISMA Extension for Scoping Review (PRISMA‐ScR) standards. Electronic databases (PubMed, Embase, and Web of Science) were searched up to October 2025. Studies evaluating miRNA expression in peri‐implantitis using human or relevant experimental samples (peri‐implant crevicular fluid [PICF], saliva, gingival tissue, serum) were included. Data were extracted and synthesized narratively.

**Results:**

Eleven studies published between 2017 and 2025 were included. Dysregulated miRNAs were consistently associated with inflammatory and osteolytic pathways, including NF‐κB, MAPK, Wnt/β‐catenin, and RANKL signaling. Downregulation of miR‐21‐3p, miR‐150‐5p, and miR‐4484, and upregulation of miR‐155 and miR‐146a were frequently reported in peri‐implantitis. Polymorphisms such as miR‐155 rs767649 and miR‐27a‐3p rs895819 were linked to disease susceptibility. However, heterogeneity in study design, small sample sizes, and lack of longitudinal validation were common limitations.

**Conclusion:**

The mapped literature indicates that miRNA expression is altered in peri‐implant disease across several biological compartments, but the evidence remains heterogeneous and largely exploratory. Specific miRNAs should be regarded as candidate biomarkers requiring independent and prospective validation rather than established diagnostic tools. Standardized sampling and analytical methods, clearly defined disease criteria, adequately powered longitudinal cohorts, and external validation are needed before clinical translation can be assessed.

## 1. Introduction

Recent years have seen widespread acceptance of dental implants as a reliable and predictable treatment option for the replacement of missing teeth. Advancement in prosthetic design, surgical method, and implant surface technology have led to high success rate and increased patient comfort and satisfaction. Despite the advantages, biological complications can jeopardize implant longevity and clinical outcomes. Hence, peri‐implantitis has now become a significant concern in modern implantology.

Peri‐implantitis is associated with inflammation around the mucosa of the implant, eventually leading to bone loss. Clinical and radiographic examination is necessary to diagnose peri‐implant condition [1]. The World Workshop held in 2017 on the classification of periodontal and peri‐implant diseases and conditions established a standardized diagnostic criterion to classify peri‐implant health and disease [2]. Clinically, peri‐implant mucositis presents with signs of inflammation without radiographic bone loss. Peri‐implantitis, in contrast, is combined with bone loss that can be appreciated in radiographs.

Accurate diagnosis of peri‐implant disease is of paramount importance in clinical practice to ensure long‐term stability of the implant. Early diagnosis provides the clinician with an opportunity to prevent further bone loss by enabling timely intervention. Expanding research of new biomarkers will allow the monitoring of the clinical condition of the implant. Recent advances in molecular biology have created new avenues for the diagnosis of peri‐implant diseases [3].

MicroRNAs (miRNAs) are small, noncoding RNA molecules that play a key role in the post‐transcriptional regulation of gene expression. They are essential for regulation of inflammation and bone metabolism, which are the two major processes in peri‐implant pathologies. With growing evidence suggesting that the presence and severity of peri‐implant mucositis and peri‐implantitis are correlated with altered expression patterns of miRNAs, they might offer to be a promising noninvasive diagnostic tool [4–6].

The primary objective of this review was to map the available evidence on miRNA expression profiles in peri‐implantitis. Secondary objectives were to characterize the biological contexts in which differential expression has been reported, summarize reported molecular associations, identify areas of convergence or inconsistency, and highlight knowledge gaps relevant to future validation. Diagnostic, prognostic, and therapeutic implications were considered as interpretive domains rather than as independent efficacy questions.

## 2. Materials and Methods

### 2.1. Review Design and Protocol

This scoping review was conducted using the methodological framework described by Arksey and O’Malley [7] and subsequent Joanna Briggs Institute guidance for scoping reviews [8]. Reporting was aligned with the PRISMA Extension for Scoping Reviews (PRISMA‐ScR) [9].

This scoping review protocol has been registered publicly on the Open Science Framework (OSF), accessible via https://archive.org/details/osf-registrations (DOI: 10.17605/OSF.IO/HZYE4; registered October 5, 2025).

#### 2.1.1. Research Question

What evidence has been reported regarding miRNA expression profiles in peri‐implantitis? The PCC framework comprised population (individuals or relevant experimental models with peri‐implant disease), concept (miRNA expression profiles and associated molecular findings), and context (peri‐implant disease).

The primary objective of this review was to map the available evidence on miRNA expression profiles in peri‐implantitis. Secondary objectives were to characterize the biological contexts in which differential expression has been reported, summarize reported molecular associations, identify areas of convergence or inconsistency, and highlight knowledge gaps relevant to future validation. Diagnostic, prognostic, and therapeutic implications were considered as interpretive domains rather than as independent efficacy questions.

### 2.2. Eligibility Criteria

The review included primary research across a broad range of designs because the objective was to map an emerging evidence base. Eligible evidence comprised human observational or clinical studies, canine experimental peri‐implantitis models, and in vitro studies directly relevant to implant‐associated inflammatory or osteogenic biology. Human evidence included studies involving peri‐implantitis or peri‐implant mucositis and biological samples such as peri‐implant crevicular fluid (PICF), saliva, serum, or peri‐implant tissue. Experimental animal and cell‐culture evidence was retained because the original protocol included these models; their translational relevance was considered separately during synthesis. Inclusion criteria: (1) population: studies that have included Individuals with peri‐implant diseases (peri‐implantitis/mucositis) and experimental models (animal ligature models and in vitro osteogenic/inflammatory models relevant to implant contexts). (2) Concept: studies that have evaluated the miRNA linked to pre‐implantitis and have explained the mechanisms. (3) Studies that have used sample as gingival biopsy or saliva or GCF or peri‐implant sulcular fluid. Exclusion criteria: (1) patients with other underlying comorbidities, (2) studies with other samples for the evaluation of miRNA, and (3) conference paper/reviews without the primary study data.

The original eligibility framework was broad; consequently, the evidence level was treated as an explicit analytical domain rather than assuming equivalence among human, animal, and in vitro findings.

### 2.3. Sources and Database Search Strategy

A comprehensive search of electronic databases, including PubMed, Embase, and Web of Science, was conducted through October 2025. The database selection reflects the searches actually performed and should not be interpreted as exhaustive coverage of all bibliographic or gray literature sources. Because gray literature was not reported as a separate search component, the possibility of publication bias or selective dissemination cannot be excluded. These searches employed targeted keywords and MeSH terms, including “MicroRNAs,” “Circulating MicroRNA,” “peri‐implantitis,” and “Dental Implants,” with full details outlined in Table 1. Search strings were tailored to suit each database’s unique syntax that is matching the predefined inclusion criteria. A detailed search string is available in the Supporting Information. All retrieved records were imported into Rayyan (https://www.rayyan.ai/) for deduplication and screening.

**Table 1:** Search string.

Search engine	Keywords
PubMed	((“MicroRNAs”[MeSH Terms] OR “Circulating MicroRNA”[MeSH Terms]) AND (“peri‐implantitis”[MeSH Terms] OR “Dental Implants”[MeSH Terms]))
Embase	((INDEXTERMS (MicroRNAs) OR INDEXTERMS (“Circulating MicroRNA”)) AND (INDEXTERMS (peri‐implantitis) OR INDEXTERMS (“Dental Implants”)))
Web of Science	((MicroRNAs OR “Circulating MicroRNA”) AND (peri‐implantitis OR “Dental Implants”))

### 2.4. Selection of Evidence Sources

Two reviewers (Madhumitha Muruganandam and Sangeeta U. Nayak) independently screened titles and abstracts against the eligibility criteria. Full texts were assessed by the same reviewers. Disagreements were resolved through discussion, with the involvement of a third reviewer (Ashita S. Uppoor) when required. Records were managed in Rayyan software (https://www.rayyan.ai/), where duplicates were removed prior to screening, and reasons for full‐text exclusions were documented for reporting in the final scoping review alongside a PRISMA flowchart (Figure 1).

**Figure 1:**
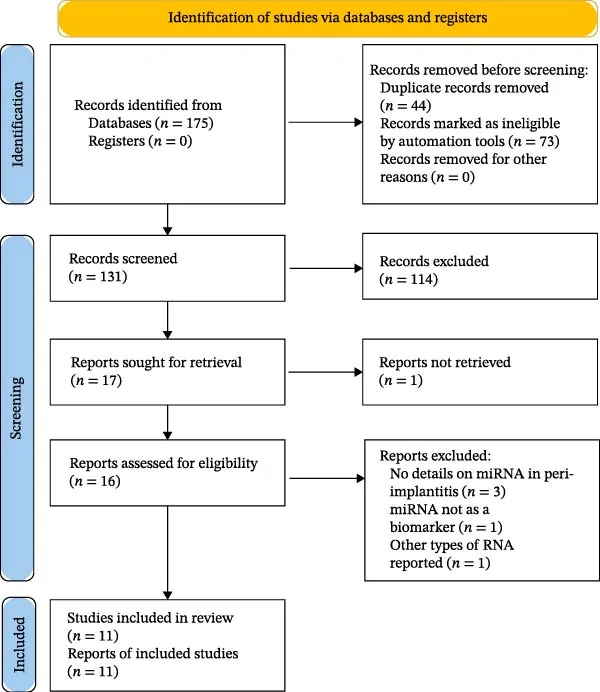
PRISMA flowchart.

### 2.5. Data Extraction and Result Synthesis

Key data from each included study was extracted and compiled by two independent reviewers using a standardized data charting form developed specifically for this review. Extracted data encompassed the author, year, study design, analyzed miRNA, sample source, extraction method or assay type, target gene, and miRNA effects, as detailed in Table 2. The underlying extracted study data were retained without alteration. Results were synthesized narratively and organized by the evidence level, expression pattern, molecular context, longitudinal/prognostic observations, and genetic associations. No meta‐analysis or pooled diagnostic estimate was performed.

**Table 2:** miRNA analysis in the included studies.

Authors [references]	Year	Design	Sample	Biological sample	miRNAs	Method/assay	Major mapped finding
Chaparro et al. [10]	2021	Exploratory study	54 patients; healthy, mucositis, peri‐implantitis	PICF	miR‐21‐3p, miR‐150‐5p, miR‐26a‐5p	miRNeasy; Qubit; qRT‐PCR	Downregulation of miR‐21‐3p and miR‐150‐5p
Urvasizoglu et al. [11]	2021	Observational case–control	Human clinical samples	Saliva	miR‐4484, miR‐4472	TaqMan microRNA assays; qPCR	miR‐4484 downregulated; miR‐4472 initially identified as upregulated
Fu et al. [12]	2025	Case–control	Human serum samples	Serum	miR‐155	TRIzol; NanoDrop; RT; SYBR Green	Upregulation of miR‐155; rs767649 associated with susceptibility
Hamed and Abdulbaqi [13]	2024	Case–control	Human peri‐implant samples	Tissue	miR‐146a, miR‐155	Stem‐loop RT; real‐time PCR	Upregulation of miR‐146a and miR‐155
Zainal Abidin et al. [14]	2025	Cross‐sectional	Human peri‐implant samples	PICF	miR‐98, miR‐145, miR‐146a, let‐7e	miRNeasy Serum/Plasma Advanced Kit	let‐7e investigated in relation to inflammatory response
Gao and Ha [15]	2025	Case–control	Human clinical samples	Not specified	miR‐27a‐3p; RUNX1	Extraction; RT‐PCR	Marked decrease in miR‐27a‐3p levels; polymorphism association
Wu et al. [16]	2017	Experimental animal study; canine ligature‐induced model	Canine model	Gingival tissue	let‐7g, miR‐27a, miR‐29a, miR‐142, miR‐145	qRT‐PCR; TargetScan/miRanda; GO/KEGG	let‐7g, miR‐27a and miR‐145 dysregulated; miR‐27a mainly downregulated and miR‐145 upregulated
Wu et al. [17]	2019	Experimental animal study; canine ligature‐induced model	Canine model	Gingival tissue	miR‐27a	qRT‐PCR	DKK2 and SFRP1 identified as direct targets; miR‐27a underexpressed
Yamamura et al. [18]	2022	In vitro experimental study	MC3T3‐E1 cultured cells	Cultured cells	miR‐155‐5p	TRIzol; 3D Gene mouse miRNA oligo chip; bioanalyzer	Increased miR‐155‐5p with osteoblastic differentiation; Runx2 and Col1a1 implicated
Tastan Eroglu et al. [19]	2024	Cross‐sectional	87 implant sites	PICF	miR‐27a	RT‐PCR	Upregulation of miR‐27a; positive correlation with keratinized mucosa width
Menini et al. [20]	2021	Prospective clinical trial; 5‐year follow‐up	7 patients, 14 implants	PICF	66 altered miRNAs; 14 overlapping with tissue	miRNeasy; Agilent Human miRNA Microarray 21.0; Qubit	Altered expression associated with >1 mm bone resorption at 5 years

### 2.6. Methodological Limitations of Evidence

Consistent with the methodological framework for scoping reviews proposed by Arksey and O’Malley [7] and further guidance from the Joanna Briggs Institute, formal quality appraisal, or risk of bias, was not undertaken because the purpose of this scoping review was to comprehensively map the existing literature and identify evidence gaps rather than evaluate the methodological quality of included studies or inform clinical effectiveness recommendations. Instead, the methodological characteristics relevant to interpretation were considered across study design, sample size, cross‐sectional versus longitudinal structure, biological sample source, RNA extraction and quantification methods, miRNA panel breadth, disease definition, population characteristics, follow‐up, confounding, and external validation. This approach is intended to contextualize the mapped evidence rather than to assign post hoc quality scores.

### 2.7. Research Settings

The scoping review was conducted in the Department of Periodontology, Manipal College of Dental Sciences, Mangalore, affiliated with Manipal Academy of Higher Education, Manipal, Karnataka, India.

## 3. Results

### 3.1. Search Results

Comprehensive database searches were conducted to begin the study selection process, providing a total of 175 articles. After 75 records were flagged for duplicate identification, 44 were immediately removed, and 29 were subjected to manual review; this left 131 articles that could be screened for titles and abstracts. 114 of these were excluded because they did not meet the requirements for inclusion.

Following this, 17 records were targeted for full‐text retrieval; 16 were assessed in full text. Full‐text exclusion criteria included reports focused on alternative RNA forms (*n* = 1), cases where miRNA had no biomarker function (*n* = 1), and studies lacking specific data on miRNA associated with peri‐implantitis (*n* = 3). Eleven articles ultimately met all inclusion criteria and served as the foundation for this review (Figure 1).

### 3.2. Characteristics of Included Evidence (Table 2)

Eleven studies were included: eight human studies, two canine experimental studies, and one in vitro study. Five studies used case–control designs, two were cross‐sectional, one was a prospective clinical trial with a 5‐year follow‐up, two were canine experimental studies, and one was an in vitro study. Human biological matrices included PICF, saliva, serum, and peri‐implant tissue; the in vitro study used cultured cells. The evidence therefore represents distinct biological levels and should not be interpreted as a single homogeneous clinical evidence base.

### 3.3. Human Clinical Evidence

The human studies demonstrated differential miRNA expression across several biological compartments. In PICF, Chaparro et al. [10] reported downregulation of miR‐21‐3p and miR‐150‐5p in peri‐implantitis relative to peri‐implant mucositis, while Zainal Abidin et al. [14] investigated miR‐98, miR‐145, miR‐146a, and let‐7e in a cross‐sectional setting. Urvasizoglu et al. [11] reported reduced salivary miR‐4484 in the context of early peri‐implantitis detection. Hamed and Abdulbaqi [13] identified increased miR‐146a and miR‐155 expression in affected peri‐implant tissue, and Fu et al. [12] reported an association between miR‐155 and the rs767649 polymorphism. Gao and Ha [15] reported decreased miR‐27a‐3p levels in association with polymorphism‐related susceptibility. Tastan Eroglu et al. [19] reported increased miR‐27a in PICF and a positive correlation with keratinized mucosa width.

### 3.4. Animal and In Vitro Evidence

The two canine studies provide preclinical evidence regarding miRNA expression and potential molecular mechanisms. Wu et al. [16] identified dysregulation of let‐7g, miR‐27a, and miR‐145 and reported enrichment of MAPK, NF‐κB, cytokine receptor, TNF, and Toll‐like receptor pathways. In a subsequent canine study, miR‐27a was underexpressed, and DKK2 and SFRP1 were identified as direct targets relevant to Wnt signaling and reosseointegration [17]. These findings support mechanistic hypotheses but cannot be assumed to represent human clinical expression patterns. The in vitro study by Yamamura et al. [18] linked increased miR‐155‐5p expression with osteoblastic differentiation of MC3T3‐E1 cells and Runx2/Col1a1 expression on titanium surfaces. This cell‐based observation is mechanistically informative but has limited direct clinical translatability.

### 3.5. Differential Expression and Biological Pathways

Across the mapped studies, miR‐21‐3p, miR‐150‐5p, and miR‐4484 were reported as downregulated in specific human clinical contexts, whereas miR‐155 and miR‐146a were reported as upregulated in the peri‐implant tissue or serum. miR‐27a showed discordant directionality across models and samples: it was underexpressed in the canine studies and in the Gao and Ha [15] human study but upregulated in the PICF‐based cross‐sectional study by Gao and Ha [15], Wu et al. [16, 17], Yamamura et al. [18], and Tastan Eroglu et al. [19]. This discordance is significant and argues against the treatment of a single miRNA as having a fixed disease‐direction signature across biological compartments. Molecular associations mapped included NF‐κB‐related inflammatory signaling, MAPK signaling, Wnt/β‐catenin signaling, osteogenic pathways, and RANKL‐related bone‐remodeling processes. Some target relationships were experimentally supported in individual studies, while other pathway assignments were derived from prediction or enrichment analyzes. It is important to distinguish between these levels of evidence.

### 3.6. Longitudinal and Prognostic Evidence

Menini et al. [20] provided the only explicitly longitudinal clinical evidence in the included set, following seven patients and 14 implants for 5 years. The study identified 66 miRNAs altered in PICF in relation to subsequent bone resorption greater than 1 mm, with 14 miRNAs overlapping with tissue findings [20]. Although this provides an important longitudinal signal, the very small sample and broad microarray profile require independent replication before these findings can be interpreted as validated prognostic signatures.

### 3.7. Genetic Polymorphism Evidence

Two included studies addressed genetic variation. Fu et al. [12] investigated the miR‐155 rs767649 polymorphism and its association with peri‐implantitis susceptibility, while Gao and Ha [15] investigated Page 6 miR‐27a‐3p polymorphisms. These studies represent genetic association evidence rather than direct evidence that circulating or tissue miRNA expression can diagnose disease. Their clinical relevance therefore requires replication across independent populations and assessment of potential confounding (Table 3).

**Table 3:** Heterogeneity in included studies.

Domain	Observed range in included evidence	Interpretive implication
Study design	Case–control (5); cross‐sectional (2); prospective clinical trial (1); animal model (2); in vitro (1)	Different designs answer different questions and vary in susceptibility to selection and temporal bias
Sample size	7 patients/14 implants to 87 implant sites; most studies <60	Small samples reduce precision and increase the likelihood that candidate signals will not replicate
Biological matrix	PICF, saliva, serum, peri‐implant tissue, cultured cells	Expression values are compartment‐specific and cannot be assumed to be interchangeable
RNA/assay approach	miRNeasy, TRIzol, TaqMan, qRT‐PCR/RT‐PCR, microarray	Extraction and analytical platforms may affect sensitivity, detectable panels, normalization, and comparability
Panel breadth	Single miRNAs to 66 miRNAs	A signal identified by a narrow targeted assay cannot be directly compared with a broad discovery panel
Follow‐up	Single time point to 5 years	Longitudinal evidence is sparse; most findings are associative rather than predictive
Evidence level	Human, canine, in vitro	Mechanistic observations from experimental models have different translational relevance from clinical observations

### 3.8. Methodological Heterogeneity

The biological matrix is particularly important. PICF, saliva, serum, tissue, and cultured cells represent different biological compartments, with distinct sources, stability characteristics, and inflammatory contexts. Consequently, a decrease in miR‐21‐3p or miR‐150‐5p in PICF cannot be directly compared with an increase in serum miR‐155 as though both represent the same biological signal [10, 12]. Likewise, the use of microarray discovery platforms versus targeted qRT‐PCR creates differences in the number of miRNAs interrogated and the analytical characteristics of the measurements. These differences constrain direct comparison and external validity.

## 4. Discussion

This scoping review mapped 11 studies investigating miRNA expression in peri‐implant disease across human, canine, and in vitro evidence. The central finding is not the establishment of a single reproducible diagnostic signature but rather the presence of a diverse set of disease‐associated miRNA observations distributed across biological compartments and experimental settings. This distinction is important because the scoping review methodology is intended to characterize the extent and nature of evidence, while the current evidence base remains too heterogeneous to support a pooled clinical estimate [7–9].

### 4.1. Convergence and Inconsistency Across miRNA Findings

Several miRNAs recur across the evidence base, particularly miR‐155, miR‐146a, miR‐27a, miR‐21‐3p, miR‐150‐5p, and miR‐4484. However, recurrence should not be interpreted as replication unless the direction, biological matrix, disease definition, population, and assay are sufficiently comparable. The clearest example is miR‐27a, for which downregulation was reported in canine peri‐implantitis models and in the Gao and Ha [15] human study, whereas upregulation was reported in the PICF study by Gao and Ha [15], Wu et al. [16, 17], Yamamura et al. [18], and Tastan Eroglu et al. [19]. This discrepancy may reflect the biological compartment, species, disease stage, assay methodology, or population differences. The current evidence does not allow for these explanations to be disentangled.

### 4.2. Disease‐Definition Heterogeneity

Differences in peri‐implantitis definitions may also contribute to apparently discordant miRNA profiles. The 2017 World Workshop provided a contemporary framework emphasizing clinical inflammation together with progressive supporting bone loss [2]. Several included studies were published before or close to this classification era, and the supplied extraction does not establish that identical diagnostic criteria were applied across all studies. Differences in case definition, radiographic thresholds, implant history, and comparator selection could therefore influence which biological phenotype was captured. Future miRNA studies should be prespecified and report standardized disease criteria so that molecular findings can be interpreted against comparable clinical phenotypes.

### 4.3. Methodological Limitations of the Evidence Base

The evidence base is dominated by small observational studies. The included sample sizes range from seven patients in the longitudinal Menini et al. [20] study to 87 implant sites in the Tastan Eroglu et al. [19] study, with most studies enrolling fewer than 60 participants or sites. Small samples increase uncertainty and make independent replication essential. Cross‐sectional and case–control designs can identify associations but cannot establish whether an altered miRNA precedes disease, reflects established inflammation, or changes as a consequence of tissue destruction. Preanalytical and analytical heterogeneity further limit comparability. PICF is a low‐volume sample, and the original manuscript notes that only seven patients in the Menini et al. [20] study met the RNA quantity threshold for microarray analysis. Differences in collection, extraction, normalization, targeted qRT‐PCR versus microarray profiling, and the breadth of miRNA panels may alter the observed signal. In addition, predicted pathway relationships should be distinguished from experimentally validated targets, particularly in animal and in vitro studies.

### 4.4. Clinical Applicability Remains Uncertain

The mapped evidence does not yet establish that miRNAs can be deployed as chairside diagnostic tests. Clinical translation would require analytical reproducibility, standardized sample collection, validated normalization procedures, defined detection thresholds, acceptable cost and turnaround time, and prospective external validation. Biological variability is also relevant: smoking, previous periodontitis, systemic diseases, medications, implant characteristics, inflammatory status, and intraindividual variation may influence miRNA profiles. These factors were not uniformly controlled or reported across the included studies. The small prospective study by Menini et al. [20] is particularly informative because it linked altered PICF miRNAs with future bone resorption, but its sample consisted of only seven patients and requires replication. Similarly, the diagnostic performance observed in individual exploratory studies should not be treated as established clinical accuracy. The current evidence therefore supports consideration of specific miRNAs as candidate biomarkers requiring external validation rather than clinical implementation.

### 4.5. Relationship to Broader Biomarker Literature

The interpretation of miRNA findings is consistent with the broader peri‐implant biomarker literature, in which multiple biological markers have been investigated, but heterogeneity in sampling, disease definitions, and analytical methods remains a major barrier to clinical translation. A 2025 systematic review and meta‐analysis of PICF biomarkers similarly concluded that the diagnostic and monitoring value of individual biomarkers remains incompletely established [4]. The present review adds a focused map of miRNA‐specific evidence and highlights the additional challenge that miRNAs may vary according to the tissue compartment and molecular pathway.

### 4.6. Strengths and Limitations of This Review

Strengths include a clearly defined evidence‐mapping objective, duplicate screening and data charting, explicit separation of human, animal, and in vitro evidence, and preservation of the original extracted study data. The search strategies for the three databases are reported, and the review was protocol‐registered and aligned with PRISMA‐ScR [7–9]. Limitations include the restriction to three bibliographic databases, the absence of a reported gray literature search, heterogeneity of eligible evidence, and the inability to perform quantitative synthesis. The broad eligibility framework also increases clinical heterogeneity, although this was addressed by separating evidence levels during synthesis.

## 5. Future Perspectives

### 5.1. Supported by the Current Evidence

• miRNA expression is altered in peri‐implant disease across PICF, saliva, serum, and peri‐implant tissue in the included studies.
• Several inflammatory and bone‐remodeling pathways recur across the mapped evidence, including NF‐κB, MAPK, Wnt/β‐catenin, and RANKL‐related signaling.
• Longitudinal evidence exists but is currently limited to one small prospective clinical study, which supports further investigation rather than clinical adoption.
• Genetic associations involving miR‐155 and miR‐27a‐3p warrant replication in independent populations.

### 5.2. Hypotheses Requiring Future Research

• Prospective, adequately powered cohorts should test whether predefined miRNA signatures predict incident or progressive peri‐implantitis after adjustment for clinical and patient‐level confounders.
• Standardized PICF, saliva, serum, and tissue collection and processing protocols are needed before inter‐study expression thresholds can be meaningfully compared.
• External validation should precede any consideration of multiplex or point‐of‐care miRNA testing. Analytical performance, cost, turnaround time, and intraindividual variability should be evaluated prospectively.
• miRNA mimics, inhibitors, therapeutic modulation, AI‐assisted prediction, and personalized treatment are reasonable research hypotheses, but they remain experimental concepts in the context of the evidence mapped here and should not be presented as established clinical interventions.

## 6. Conclusion

This scoping review mapped 11 studies examining miRNA expression in peri‐implant disease across human clinical, canine experimental, and in vitro evidence. Recurrent findings included altered expression of miR‐21‐3p, miR‐150‐5p, miR‐4484, miR‐155, miR‐146a, and miR‐27a in specific biological and experimental contexts, with reported associations involving inflammatory and osteolytic pathways. The strongest longitudinal signal came from a small 5‐year clinical study identifying 66 altered PICF miRNAs associated with subsequent bone resorption [20]. Overall, the evidence supports miRNAs as a biologically informative and hypothesis‐generating research field but not as established diagnostic or therapeutic tools. Heterogeneity in biological matrices, assay methods, disease definitions, study populations, sample sizes, and follow‐up currently limits direct comparison and external validity. Future research should prioritize standardized methodology, prospective longitudinal validation, independent replication, and clinically relevant performance evaluation before any diagnostic or therapeutic application is considered.

**Nomenclature**
AI: Artificial intelligence
GO: Gene Ontology
KEGG: Kyoto Encyclopedia of Genes and Genomes
KMW: Keratinized mucosa width
MAPK: Mitogen‐activated protein kinase
miRNA: MicroRNA
NF‐κB: Nuclear factor kappa B
PCC: Population, concept, and context
PICF: Peri‐implant crevicular fluid
qRT‐PCR: Quantitative reverse transcription polymerase chain reaction
RANKL: Receptor activator of nuclear factor kappa‐B ligand
RT‐PCR: Reverse transcription polymerase chain reaction
RUNX1: Runt‐related transcription factor 1
SMAD: Mothers against decapentaplegic homolog
TLR: Toll‐like receptor
Wnt: Wingless‐related integration site signaling pathway.

## Author Contributions

**Madhumitha Muruganandam**: conceptualization, methodology, software, validation, formal analysis, investigation, resources, data curation, writing – original draft. **Sangeeta U. Nayak**: methodology, software, investigation, supervision, project administration. **Ashita S. Uppoor**: visualization, supervision, project administration. **Arpit Gupta**: data curation, writing – original draft, writing – review and editing, visualization. **Santhosh Kumar**: writing – review and editing, visualization.

## Funding

This scoping review did not receive any external funding.

## Ethics Statement

Ethical clearance was not applicable to the present review as it was conducted as a scoping review based exclusively on previously published literature and did not involve human participants or the collection of primary data.

## Conflicts of Interest

The authors declare no conflicts of interest.

## Supporting Information

Additional supporting information can be found online in the Supporting Information section.

## Supporting information


**Supporting Information** The Supporting Information includes the completed PRISMA‐ScR checklist, which provides a detailed mapping of the reporting items to the corresponding sections of the manuscript. The checklist is provided to facilitate transparency and demonstrate adherence to the PRISMA extension for Scoping Reviews (PRISMA‐ScR) reporting guidelines.

## Data Availability

The authors confirm that the data supporting the findings of this study are available within the article and its Supporting Information.
