# Supplementary material for: Mapping the Expression Profile of MicroRNA in Peri‐Implantitis: A Scoping Review
Source: Int J Dent. 2026 Oct 21;2026:7830337. doi: 10.1155/ijod/7830337 (PMC13685660; doi:10.1155/ijod/7830337)
Supplement: Supplementary file 1 [file IJOD-2026-7830337-s001.docx]

**PRISMA-ScR CHECKLIST**

Mapping microRNA expression profiles in peri-implantitis: A scoping review

| **Item** | **Section** | **PRISMA-ScR requirement** | **Manuscript location** | **Status** |
| --- | --- | --- | --- | --- |
| 1 | Title | Identify the report as a scoping review. | Title page | Reported |
| 2 | Structured summary | Provide a structured summary including background, objectives, eligibility criteria, sources, charting methods, results, and conclusions. | Abstract | Reported; eligibility/charting summarized. |
| 3 | Rationale | Describe the rationale in the context of existing knowledge. | Introduction | Reported |
| 4 | Objectives | Provide an explicit statement of questions/objectives using key elements. | Introduction; Methods 2.1 | Reported |
| 5 | Protocol and registration | Indicate whether a protocol exists and provide registration information. | Methods 2.1 | Reported; OSF DOI supplied. |
| 6 | Eligibility criteria | Specify inclusion/exclusion criteria and how studies were grouped for synthesis. | Methods 2.2 | Reported; evidence levels explicitly separated. |
| 7 | Information sources | Describe databases/registers and dates searched. | Methods 2.3 | Reported for PubMed, Embase, Web of Science through Oct 2025. |
| 8 | Search | Present full electronic search strategy for at least one database. | Methods 2.3; search table | Reported for all three searched databases. |
| 9 | Selection of sources of evidence | State methods for selecting evidence and whether independently/duplicate. | Methods 2.4 | Reported |
| 10 | Data charting process | Describe methods of data charting and whether independently/duplicate. | Methods 2.5 | Reported |
| 11 | Data items | List and define all variables for which data were sought. | Methods 2.5 | Reported |
| 12 | Critical appraisal of individual sources | If done, describe methods; if not, state that no formal appraisal was conducted. | Methods 2.6 | No formal risk-of-bias appraisal conducted; structured methodological contextualization used. |
| 13 | Synthesis of results | Describe methods of handling/charting results and present synthesis. | Methods 2.5; Results 3 | Narrative synthesis reported. |
| 14 | Selection of sources | Give numbers screened, assessed, and included with reasons for exclusions. | Results 3.1 | Reported; one unretrieved/not-assessed full text requires author confirmation. |
| 15 | Characteristics of sources | For each source, present characteristics and relevant data. | Results 3.2; study table | Reported |
| 16 | Critical appraisal within sources | If conducted, present results. | Results/Discussion | No formal appraisal; limitations discussed. |
| 17 | Results of individual sources | For each source, present relevant results that address the question. | Results 3.2-3.7 | Reported in study table and narrative. |
| 18 | Synthesis of results | Summarize and synthesize results across sources. | Results 3.3-3.8; Discussion | Reported |
| 19 | Summary of evidence | Summarize main results, confidence/limitations, and relevance. | Discussion; Conclusion | Reported; quantitative certainty assessment not performed. |
| 20 | Limitations | Discuss limitations of the scoping review process. | Discussion 4.6 | Reported |
| 21 | Conclusions | Provide general interpretation and implications/future research. | Future perspectives; Conclusion | Reported with cautious translational framing. |
| 22 | Funding | Describe sources of funding. | Statements | No external funding reported. |
| 23 | Conflicts of interest | Declare competing interests. | Statements | No conflict reported. |
| 24 | Availability of data | State availability of data/charting forms/materials if applicable. | Statements | Data availability statement included. |
